# Additive Manufacturing Polyurethane Acrylate via Stereolithography for 3D Structure Polymer Electrolyte Application

**DOI:** 10.3390/gels8090589

**Published:** 2022-09-15

**Authors:** Muhammad Faishal Norjeli, Nizam Tamchek, Zurina Osman, Ikhwan Syafiq Mohd Noor, Mohd Zieauddin Kufian, Mohd Ifwat Bin Mohd Ghazali

**Affiliations:** 1 SMART RG, Faculty of Science and Technology, Universiti Sains Islam Malaysia, Nilai 71800, Malaysia; faishal5453@raudah.usim.edu.my; 2 Department of Physics, Faculty of Science, Universiti Putra Malaysia, Serdang 43400, Malaysia; nizamtam@upm.edu.my; 3 Centre for Ionics Universiti Malaya, Department of Physics, Faculty of Science, Universiti Malaya, Kuala Lumpur 50603, Malaysia; zurinaosman@um.edu.my (Z.O.); mzkufian@um.edu.my (M.Z.K.); 4 Physics Division, Centre of Foundation Studies for Agricultural Science, Universiti Putra Malaysia, Serdang 43400, Malaysia; imnoor@upm.edu.my

**Keywords:** additive manufacturing, 3D printing, polyurethane acrylate, ionic conductivity, gel polymer electrolyte

## Abstract

Additive manufacturing (AM), also known as 3D-printing technology, is currently integrated in many fields as it possesses an attractive fabrication process. In this work, we deployed the 3D-print stereolithography (SLA) method to print polyurethane acrylate (PUA)-based gel polymer electrolyte (GPE). The printed PUA GPE was then characterized through several techniques, such as Fourier transform infrared (FTIR), electrochemical impedance spectroscopy (EIS), X-ray diffraction analysis (XRD), thermogravimetric analysis (TGA), differential scanning calorimetry (DSC) and scanning electron microscope (SEM). The printed GPE exhibited high ionic conductivity of 1.24 × 10^−3^ S cm^−1^ at low-lithium-salt content (10 wt.%) in ambient temperature and favorable thermal stability to about 300 °C. The FTIR results show that addition of LiClO_4_ to the polymer matrix caused a shift in carbonyl, ester and amide functional groups. In addition, FTIR deconvolution peaks of LiClO_4_ show 10 wt.% has the highest amount of free ions, in line with the highest conductivity achieved. Finally, the PUA GPE was printed into 3D complex structure to show SLA flexibility in designing an electrolyte, which could be a potential application in advanced battery fabrication.

## 1. Introduction

In recent decades, the advances in electric vehicles and portable electronic devices have spurred demand in the development of advanced energy storage technologies that are capable of improving energy and power densities, life cycle and design flexibility. Generally, batteries are one of the best choices for energy storage, as they have high energy density, long cycle life, highly reliable, portable and economical price [1]. The performance of the batteries is heavily dependent on the intrinsic material properties of the electrodes and electrolyte and the structure of the devices.

The research and development of energy storage technologies among academia and industry has accelerated during the last two decades. However, most academic reviews and research that have been conducted only focus on material advancement for better battery performance. Little attention has been paid to the fabrication process, which serves as the way to transform material into a device or product. Traditional fabrication methods of electrodes and solid-state electrolytes have problems with controlling the battery component geometry and architecture [2]. Moreover, conventional battery 2D design needs a bigger footprint area to produce higher battery capacity [3]. However, the rising desire for smaller, lighter and portable electronics has increased the demand for high-capacity and efficient energy storage devices with smaller footprints.

The 3D structural electrolytes offer larger available interphase surface area and shorter ion diffusion pathways compared to conventional 2D planar electrolytes [4]. In addition, a 3D structural solid electrolyte is one of the ways to make a 3D-structured solid-state battery that could be useful for space optimization in electronics devices. It could eliminate battery shape consideration in designing an electronic device product, such as smartphones, laptops, watches, etc. Hence, additive manufacturing (AM), also referred to as 3D printing, is one of the techniques that can be utilized to produce a flexible structural design of any object, including electrolytes.

Additive manufacturing printing is a technology that allows for the rapid and accurate fabrication of complex 3D architecture. Further, 3D-printing technology has enabled the production of low-volume, customized objects with complicated geometries, utilizing materials with equivalent or better qualities to those produced through conventional manufacturing [5], thus, offering numerous scopes in applied sectors. These qualities have piqued the interest of many researchers to integrate 3D printing into energy storage fabrication. Lithium-ion batteries (LIBs) could be 3D printed in any shape, allowing the battery form factor to be adjusted to meet a specific product design. This would also make it easier to employ the battery as a structural component, as well as improve the battery’s energy and power density.

Numerous studies related to 3D-print battery components, which are anode, cathode and electrolyte, have been conducted, including direct ink writing (DIW) [6,7], fused deposition modelling (FDM) [8], electrophoretic deposition (EPD) [9,10], laminated object manufacturing (LOM) and stereolithography (SLA) [11,12]. DIW is a 3D-printing technique that is mostly used to construct 3D batteries. However, DIW depends on the rheological properties of the inks and needs a post-process, such as freeze drying and sintering, before obtaining the final product. Nevertheless, SLA is a technology in which a three-dimensional object is produced layer by layer by utilizing photopolymerization to harden each layer of photo-polymer resins. This technology does not involve complex material preparation for the printable inks or complex post-treatment of printed items. In contrast to other printing methods, SLA can create a real 3D architecture with high efficiency, low cost and high-resolution printing [2]. As a result, SLA can reduce interfacial impedance while also increasing the mass loading of active materials [11]. However, the implementation of SLA in printing electrolytes lacks attention from researchers, even though the technology has a lot of potential.

Basically, SLA or other additive manufacturing categories have three main components: materials, 3D printer and computer-aided design (CAD). Any adjustments to these components could change the characteristics or performance of the final 3D-printed products. For now, the material for SLA is limited as it needs to be a photopolymer for the process. Poly(urethane acrylate) or PUA is a widely used photopolymer material and it also can be used to fabricate polymer electrolytes as there were several studies that deployed PUA as the polymer matrix for the polymer electrolyte [13,14,15]. It also possesses a high ionic conductivity, excellent mechanical strength and physically tunable characteristics [16].

In this work, 3D-printed gel polymer electrolytes were fabricated using PUA as the polymer host via the SLA technique. The commercially available PUA-based resin used in this work was incorporated with lithium perchlorate (LiClO_4_), dissolved in dimethylformamide (DMF). The effect of salt concentrations on the conductivity, dielectric properties, structural properties and thermal stability of the 3D-printed GPEs were studied.

## 2. Results and Discussion

### 2.1. X-Ray Diffraction Analysis (XRD)

**Figure 1:**
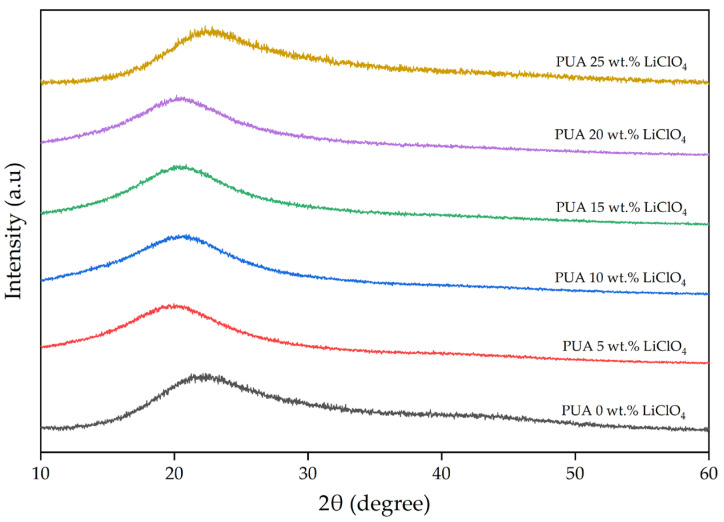
XRD patterns of 3D-printed PUA-LiClO_4_-based GPE system.

The purpose of the XRD analysis was to investigate the crystallinity behavior of polymer materials. Figure 1 shows the recorded XRD pattern of 3D-printed PUA-based GPE with different concentrations of LiClO_4_. There were broad humps within 15° to 30° observed on the XRD pattern of the PUA electrolytes. The absence of crystalline peaks on the recorded 3D-printed PUA-based GPE XRD patterns indicated the samples are amorphous. In addition, no peaks corresponding to LiClO_4_ were detected in the pattern, suggesting a complete dissolution of LiClO_4_ in PUA electrolytes [17]. The addition of 5 wt.% LiClO_4_ into the PUA sample shifted the peak at 2θ angle of 22° to the left. Further addition of LiClO_4_ in PUA 5 wt.% LiClO^4^ shifted the maximum peak of XRD diffractogram to the right until the sample consisted of 25 wt.% LiClO_4_. The large shift in the maximum peak in PUA 25 wt.% LiClO_4_ can be attributed to the large amount of salt in the electrolyte.

**Figure 2:**
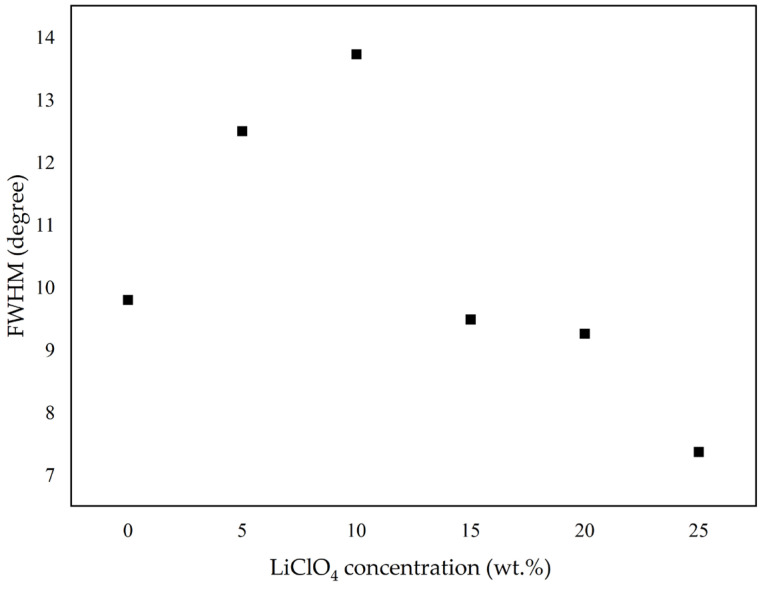
FWHM of XRD pattern for 3D-printed PUA-LiClO_4_-based GPE system.

In addition, the complexation between lithium salt and polymer matrix causes the change in free volume of polymer that results in a greater amorphous phase of the GPE. The amorphous phase in the polymer electrolyte is essential since it aids in enhancing the electrolyte’s ionic conductivity. Compared to the crystalline phase, the amorphous phase has greater ionic diffusion as the polymer chain induces quicker segmental motion and bond rotations, leading to better conductivity. The increase in the amorphous phase causes a decrease in the energy barrier to the segmental motion of the GPE. This will help ions and facilitate the ions’ movement, hence, improving the ionic conductivity [18]. Figure 2 shows the full width at half maximum (FWHM) of the XRD hump. The high value of FWHM indicates a high amorphous fraction in the polymer [19]. Based on Figure 2, the value of FWHM increased until 10 wt.% of LiClO4, then decreased starting at 15 wt.% of LiClO_4_ and beyond it. It means that the maximum amorphous region is at 10 wt.% LiClO_4_ and decreases at higher concentrations. The decrease in FWHM implies a decreasing amorphous nature of GPEs. It was observed that the value of FWHM was in agreement with the ionic conductivity pattern discussed in the EIS measurement section below, as the ionic conductivity for GPEs only increased up to 10 wt.% LiClO_4_ before going down.

### 2.2. Scanning Electron Microscopy (SEM)

**Figure 3:**
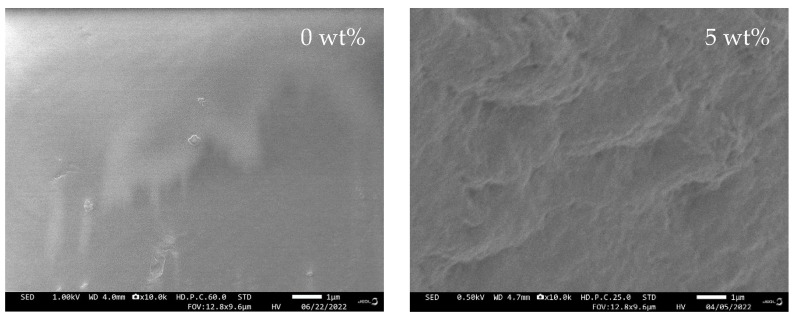

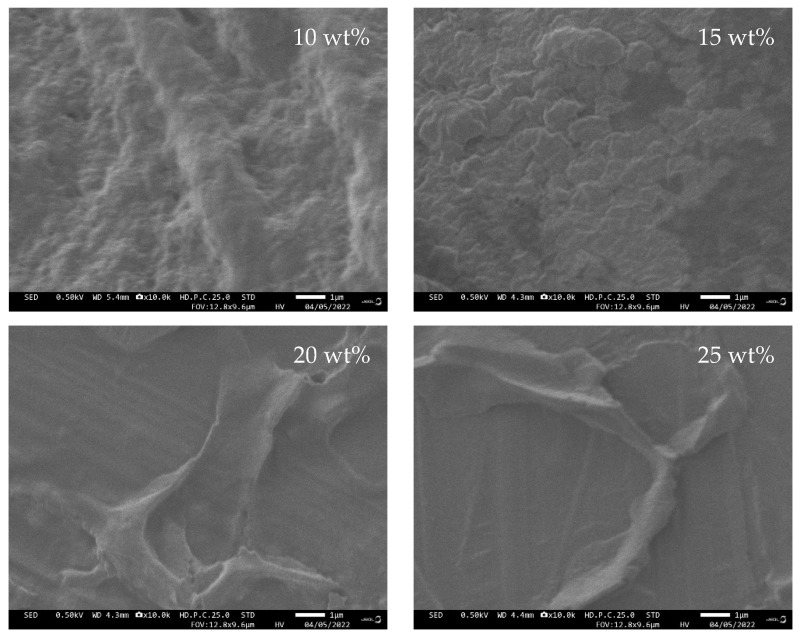
Morphology of SLA-printed PUA-based GPE with different LiClO_4_ concentrations.

Figure 3 depicts microstructures of 3D-printed PUA-based GPE with different concentrations of LiClO_4_, taken by using scanning electron microscopy (SEM). The results from the SEM are in line with the variation in ionic conductivity of the 3D-printed PUA-based GPE. The addition of LiClO_4_ salt into the GPE altered the morphology structure of the 3D-printed GPE. The morphology surface of the 3D-printed PUA changed from smooth to textured surface based on the salt concentration. This indicates the presence of structural rearrangement in the polymer chain and leads to Li^+^ cation transportation in the polymer host [20]. The 3D-printed PUA-based GPE with 10 wt.% LiClO_4_ has the roughest surface and has micro-size pores, thus, increasing the surface area for the Li^+^ cation transporting characteristic [21]. Referring to Figure 3, the same sample has higher conductivity compared to the other salt concentrations. With a further addition of salt beyond 10 wt.% LiClO_4_ to the system, the salt started to recrystallize/agglomerate due to the increasing salt concentration or might be due to the formation of ion pairs, which leads to a reduction in the ionic conductivity [22].

### 2.3. Fourier-Transform Infrared Spectroscopy (FTIR)

**Figure 4:**
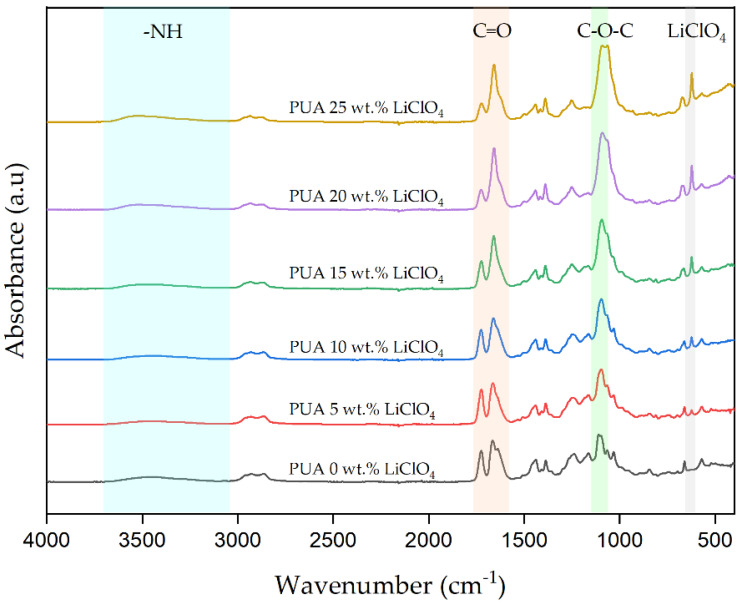
FTIR spectra of 3D-printed PUA GPE with different LiClO_4_ concentrations.

The addition of LiClO_4_ into the polymer matrix can alter the molecular interactions. The FTIR spectra of 3D-printed PUA/LiClO_4_ electrolytes are depicted in Figure 4. The changes in intensity and the shift in wavenumber for the spectrum peak indicates there are complexation between Li^+^ and PUA molecules, especially in certain functional groups in the PUA. In Figure 4, there are three main functional groups that contribute to the coordination of Li^+^, namely -NH stretching mode (3800–3100 cm^−1^), carbonyl (C=O) (1725–1650) and ester group (C-O-C) (1300–1000). Both of the oxygen and nitrogen atoms have excessive electrons that are able to interact with the lithium cations dissociated from LiClO_4_ [23].

From Figure 4, it is observed that the addition of LiClO_4_ salt into the PUA polymer host eventually shifted the -NH stretching band to a higher wavenumber. The nitrogen atoms from the N-H group in PUA formed a coordination with Li^+^ cations and made the H-bond of -NH stretching weaken and form polymer–salt complexes [24]. This causes molecules in the -NH group to now vibrate at low wavelengths, resulting in a shift in the FTIR spectrum to higher wavenumbers.

The interaction of LiClO_4_ with the carbonyl group (C=O) occurs at two wavenumbers: 1723 cm^−1^ and 1666–1658 cm^−1^. The peak at 1723 cm^−1^ corresponds to free C=O groups and 1666 to 1658 cm^−1^ can be attributed to the contribution of bound C=O groups in PUA and dipole interaction with Li^+^, respectively. The peak intensity of the 1723 cm^−1^ band as the salt content decreases might be due to the increasing number of free C=O associated with Li^+^ cations. As more Li ions interact with the oxygen atom, the C=O in the PUA chain is weakened; however, the stability of GPE is enhanced. Meanwhile, the peaks at 1666–1658 cm^−1^ intensified and changed to a lower wavenumber, implying that the carbonyl group interacted more with lithium salts, weakening the C=O bond and allowing the Li^+^ cation to share electron density with oxygen atoms. The oxygen ion in the carbonyl group of PUA serves as an electron donor atom, forming coordination bonds with the Li^+^ cation in the polymer host structure. As the electron density of the carbonyl group diminishes, the vibrational energy of the group decreases. As a result, the frequency of vibration shifted to a lower wavenumber [25].

The C-O-C stretching band at 1097 cm^−1^ did not show any shifting up to the sample with 10 wt.% LiClO_4_. For the sample with 15 wt.%, the peak starts to slightly shift to a lower wavenumber until it reaches 1091 cm^−1^ for the sample consisting of 25 wt.% LiClO_4_. In addition, the band intensity also increased with the increasing salt content. These conditions might occur due to the strong interaction of ether oxygen in the polymer matrix PUA with Li^+^ cations. The Li^+^ cation is capable of coordinating with the PUA polymer and weakening the C-O-C groups. The addition of LiClO_4_ salt into the polymer causes the decreasing oxygen atom electron density from the C-O-C bond and, thus, frees some of the H-bonded ether groups. These findings suggest that adding LiClO_4_ salt to PUA causes multiple interactions that could change some of the polymer’s microstructure.

The interaction between the polymer matrix and the lithium salt was further investigated using the FTIR deconvolution method. The output information of the deconvoluted absorbance peak could be contributed by the presence of free ions and ion pairs [26,27]. New absorbance peaks for free ions and ion pairs were discovered at 616 and 633 cm^−1^, respectively, after adding LiClO_4_ to the polymer electrolyte, as reported by Sim et al. [28]. In addition, Chen et al. [29] identified peaks for free ions and ion pairs at 624 cm^−1^ and 635 cm^−1^, respectively, formed from the absorbance of ClO_4_^−^. Moreover, Naiwi et al. [21], in their study, assigned 613 and 614 cm^−1^ as free ions and 633 and 634 cm^−1^ as ion pairs of ClO_4_^−^. Based on these reports, the free ions and ion pairs of LiClO_4_ can be found at wavenumbers between 650 and 600 cm^−1^.

**Figure 5:**
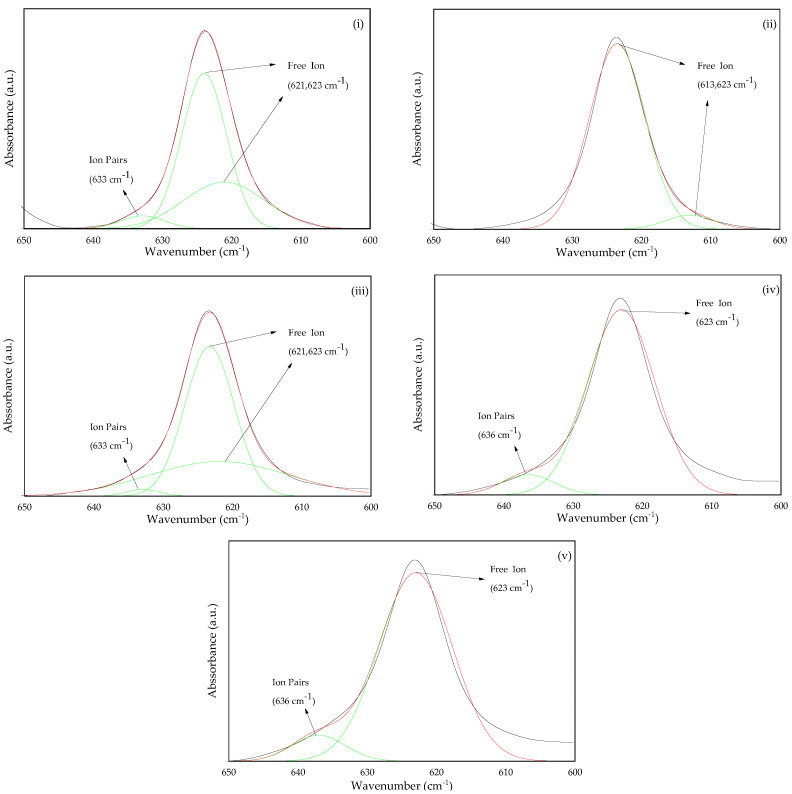
FTIR deconvolution between 650 and 600 wavenumbers for 3D-printed PUA-based GPE with (**i**) 5 wt.%, (**ii**) 10 wt.%, (**iii**) 15 wt.%, (**iv**) 20 wt.% and (**v**) 25 wt.% LiClO_4_.

The results of deconvoluted peaks are shown in Figure 5 and Table 1. In this study, the peaks at 613, 621 and 623 cm^−1^ are designated for free ions; meanwhile, peaks at 633 and 636 are for the ion pairs. The area under the graph of free ions and ion pairs was calculated using Equations (11) and (12) in order to obtain the percentage area of each species. Based on Table 1, the area percentage of free ions increased from 94.98% at 5 wt.% of LiClO_4_ content to a maximum of 99.90% at 10 wt.% of LiClO_4_ content. This is due to the increased salt content in the 3D-printed GPE, which increased the amount of free ions in the electrolyte. This shows that adding LiClO_4_ salt stimulates the formation of free ions, resulting in a greater number of charge carriers and resulting in a higher ionic conductivity. However, when the amount of LiClO_4_ exceeded 10 wt.%, the percentage of ion pairs rose from 0.01% to 9.06% at 25 wt.% of LiClO_4_ content and vice versa for the free ions. This phenomenon was caused by the preferential production of ion pairs rather than free ions when the amount of lithium salt is too high. With the higher amount of LiClO_4_, the further free ions dissociated within the same volume of the polymer matrix. At a certain level of salt content, it became saturated and the distance of two free ions got closer and combined due to the Coulomb attraction force, then became neutral. Since there are fewer free ions, there are fewer charge carriers, which will lower the ionic conductivity of polymer electrolytes.

### 2.4. Electrochemical Impedance Spectroscopy (EIS)

**Figure 6:**
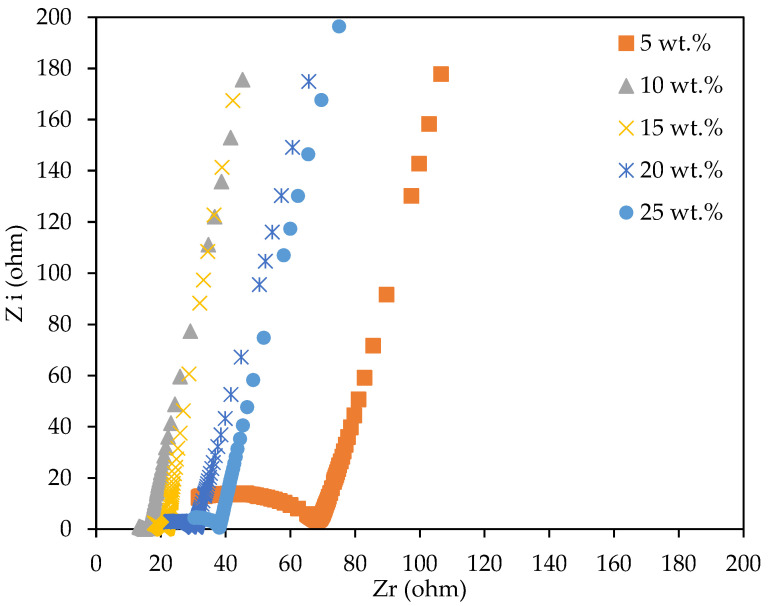
Nyquist plot for SLA-printed PUA-based GPE system.

**Figure 7:**
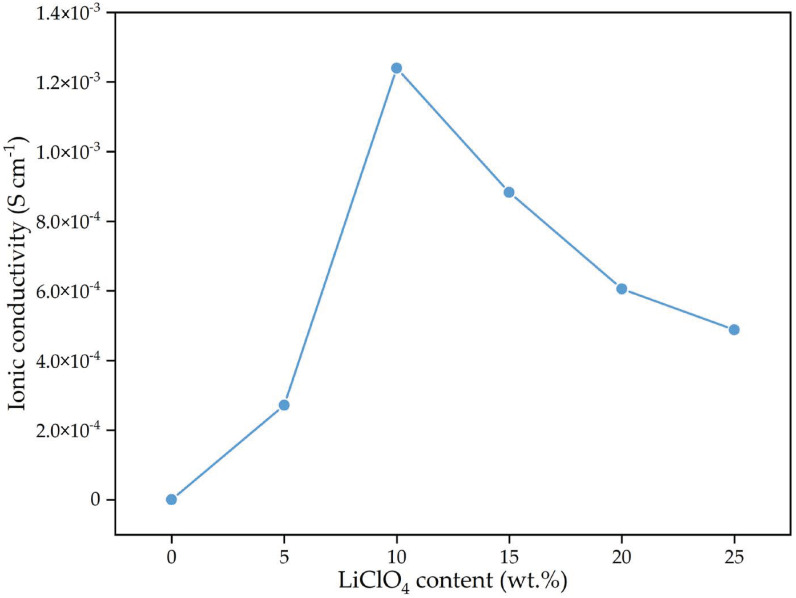
Room temperature ionic conductivity of 3D-printed PUA-based GPE with different LiClO_4_ concentrations.

The Nyquist plot depicted in Figure 6 is the combination of the real (Z_r_) and negative imaginary (Z_i_) impedance of the 3D-printed PUA-based GPE, consisting of 0 wt.% to 25 wt.% of LiClO_4_ salt content at room temperature. The interception point between depressed semicircle plot and the incline plot was determined and used to calculate the bulk resistance (R_b_) of the samples. The measured R_b_ and calculated ionic conductivity are shown in Table 2. Based on Figure 7 and Table 2, the optimum room temperature ionic conductivity was obtained at low LiClO_4_ concentrations of 10 wt.% with a value of 1.24 × 10^−3^ S cm^−1^, which is four orders of magnitude higher compared to the sample without LiClO_4_ salt (3.11 × 10^−7^ S cm^−1^). The GPE conductivity achieved was comparable to the PUA GPEs from previous studies, as shown in Table 3. The addition of LiClO_4_ more than 10 wt.% decreased the conductivity of 3D-printed PUA-based GPE due to a decrease in the number of free ions in the system. The ionic conductivity results have a similar pattern with the trend of free ion number with increasing salt content that was obtained from FTIR deconvolution (Table 1). Based on Table 1, the addition of salt increased the number of free ions up to 10 wt.% of LiClO_4_ with 99.90% free ions and decreased beyond that. This occurred because of the interaction between the quantity of charge carriers and the number of free ions [30]. The volume of free ions rose as more LiClO_4_ was added. As a result, the quantity of charge carriers increased, thus, increasing PUA GPE’s ionic conductivity. However, ionic conductivity decreased as free ions dropped at concentrations greater than 10 wt.% LiClO_4_. This occurred because of the production of ion pairs. As the number of free ions increased, the space between them shrank and eventually merged (became neutral).

Theoretically, the number density ( n) and mobility ( μ) of charge carriers determine the ionic conductivity values of electrolytes, as shown in Equation (1), where e is electron charge (constant).

(1) σ=nμe

**Figure 8:**
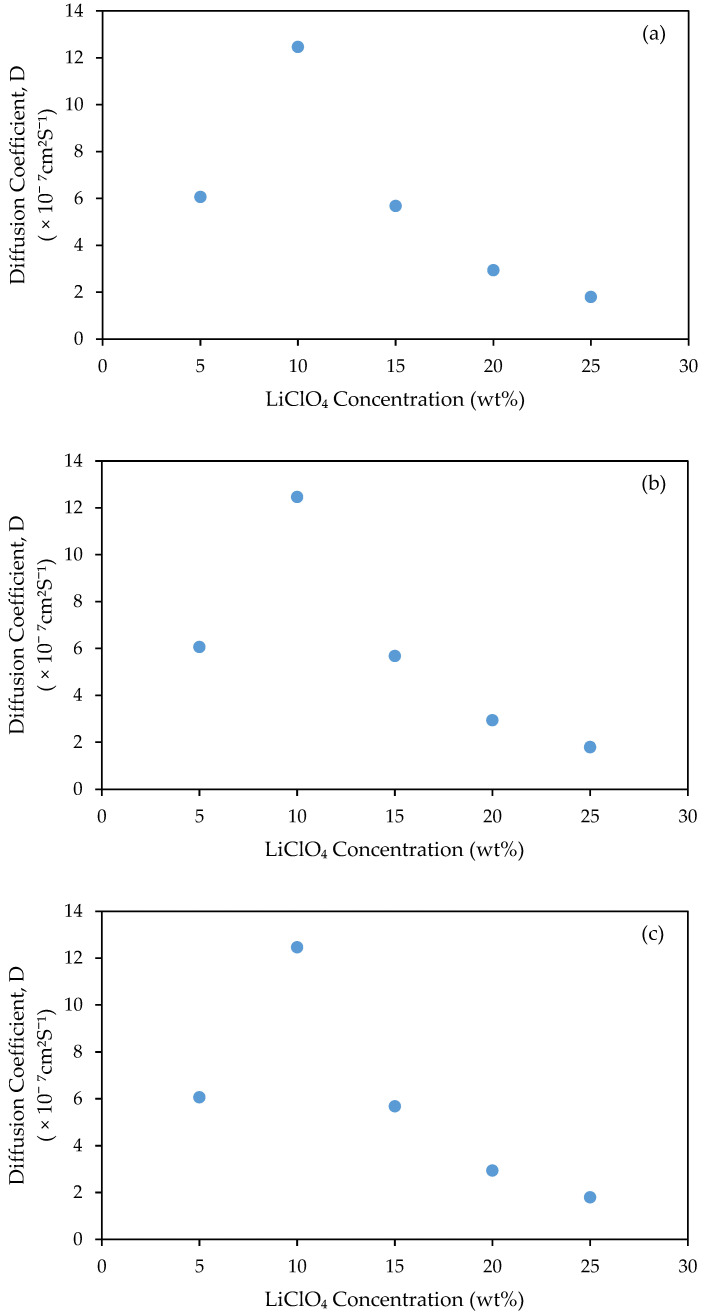
(**a**) Charge carrier number density (n), (**b**) mobility (µ) and (**c**)diffusion coefficient (D) of 3D-printed PUA-based GPE with various concentrations of LiClO_4_.

According to Figure 8a, the charge carrier density increases linearly with the addition of LiClO_4_ concentration from 7.06 × 10^23^ to 4.28 × 10^24^ cm^−3^ [31]. However, it only enhances the ionic conductivity up to samples with 10 wt.% LiClO_4_ because of the decreasing value of the mobility and diffusion coefficient of charge carrier, as shown in Figure 8b,c. The decreasing value of *μ* and D were due to ion collision [32]. The higher number density of charge carrier indicated a greater number of free ions within the GPE, causing a higher rate of collision between the ions. Hence, the collision between the ions would be easier and it slows down the mobility of the charge carrier along with the diffusion coefficient.

**Figure 9:**
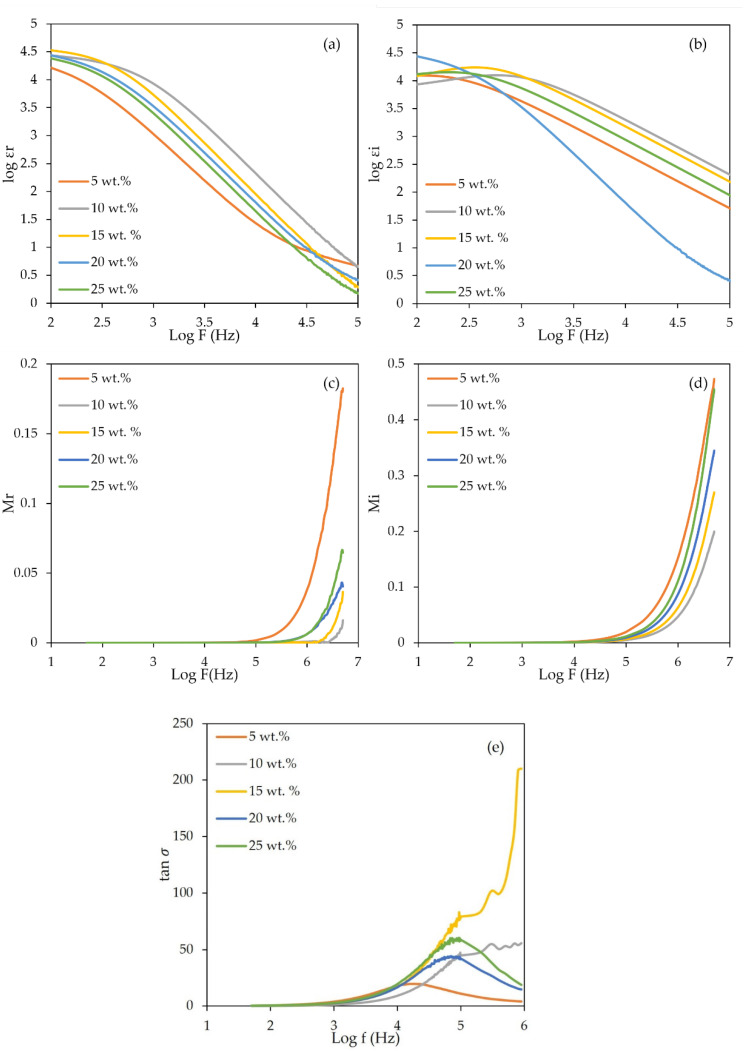
(**a**) Log dielectric constant, (**b**) log dielectric loss, (**c**) real-part electrical modulus, (**d**) imaginary-part electrical modulus and (**e**) tan δ as a function of log frequency for 3D-printed PUA-based GPE with different LiClO_4_ concentrations.

Figure 9a,b depicts a graph of dielectric constant ( $\varepsilon_{r}$ ) and dielectric loss ( $\varepsilon_{i}$ ) as a function of frequency for different weight contents in LiClO_4_ of 3D-printed PUA-based GPE at ambient temperature. The values of $\varepsilon_{r}$ and $\varepsilon_{i}$ were observed higher at low frequency, decreasing towards higher frequency. The higher values of $\varepsilon_{r}$ and $\varepsilon_{i}$ at lower frequencies were due to ion polarization [36] and space charge effect [37]. Ion polarization is a result of decreasing charge accumulation, which contributed to the decreasing value of $\varepsilon_{r}$ and $\varepsilon_{i}$ . At higher frequency, the periodic reversal of electric field takes place faster until the ions do not have enough time to diffuse in the electric field direction [38]. The 10 wt.% LiClO_4_ achieved the highest value of $\varepsilon_{r}$ and $\varepsilon_{i}$ compared to the other concentrations. Beyond the 10 wt.% LiClO_4_, the value of $\varepsilon_{r}$ and $\varepsilon_{i}$ reduced accordingly. This is because the 10 wt.% has the highest value of free ions, charge carrier mobility and diffusion coefficients; meanwhile, the values decrease beyond 10 wt.% of LiClO_4_.

The real electrical modulus (M_r_) and imaginary electrical modulus (M_i_) of different LiClO_4_ concentrations as a function of log frequency are shown in Figure 9c,d. Based on Figure 9c,d, the value of M_r_ and M_i_ increased with frequency and started increasing sharply from 5 Hz to 4 Hz, respectively. This demonstrated that the 3D-printed PUA-based GPE samples acted as ionic conductors [36] and consisted of charge carriers that play a big role in enhancing the ionic conductivity. Both M_r_ and M_i_ are close to zero at low frequency with a long tail. The long tail indicates that there was a large capacitance associated with the electrodes due to a slight polarization effect [22,24].

A graph of tan δ as a function of log frequency is shown in Figure 9e. The maximum peaks for each sample with different concentrations of LiClO_4_ salt were determined to calculate relaxation time ( τ ). The relaxation time ( τ ) demonstrates the existence of the relaxation parameter within the GPE [37,39]. From Figure 9e, the peaks are observed at high frequency and the 10 wt.% LiClO_4_ sample has the highest frequency. The higher the frequency, the more reduced the amount of relaxation time would be. Relaxation time is proportional to the time required for ions to diffuse from one electrode to another. Relaxation time is proportional to the time required for ions to diffuse between the two electrodes. The relaxation time for 3D-printed GPE is summarized in Table 4. From the table, the lowest relaxation time is 6.37 × 10^−8^ s at 10 wt.%, parallel with the highest conductivity achieved by the GPE.

### 2.5. Transference Number Measurement (TNM)

**Figure 10:**
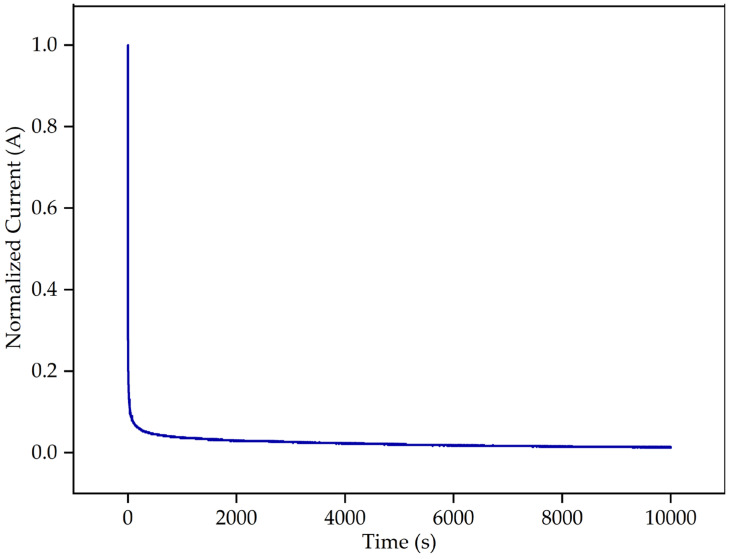
Normalized current versus time of PUA-10 wt.% LiClO_4_ GPE.

The transference number analysis of the most conductive 3D-printed GPE film was performed to evaluate the contribution of ions to the overall charge transport in the GPE system. Based on Figure 10, the normalized current decreased with time as the ionic species in the GPE being depleted until the current became stable in a fully depleted condition. The cell is polarized in the steady state and current flows due to electron movement across the electrolyte and interface. If the ionic currents across an ion-blocking electrode decline fast with time, indicating that the electrolyte is mainly ionic [40]. The transference number value of PUA-10 wt.% LiClO_4_ GPE was found to be 0.98. This value suggests that the charge carriers within the GPE are predominantly ions as the values of the ionic number were close to unity. This result is in line with previous research [41,42] that suggested the charge transport in the polymer electrolyte is primarily due to ions and electron contribution to the system was insignificant.

### 2.6. Thermogravimetric Analysis (TGA)

**Figure 11:**
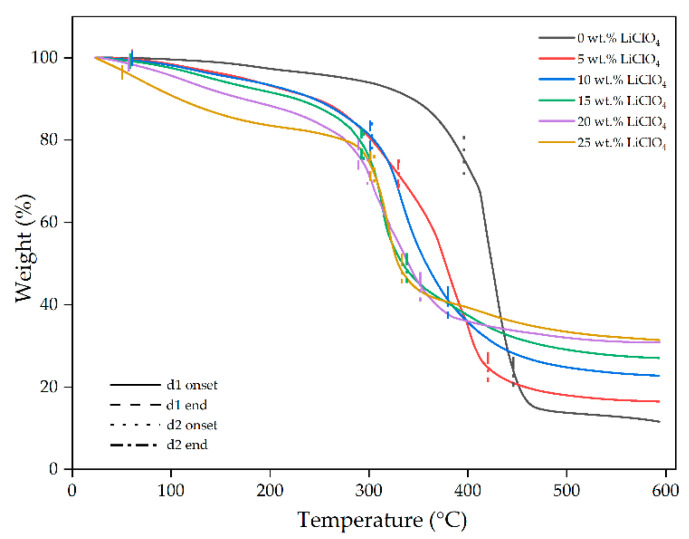
Thermograms of 3DP GPE PUA with different wt.% of LiClO_4_.

TGA is performed to evaluate the thermal stability and decomposition temperature of the 3D-printed GPE by measuring the weight loss of the sample over the increasing temperature. The TGA thermograms of 3D printed PUA GPEs with different LiClO^4^ concentration are shown in Figure 11. The decomposition of the 3D-printed GPEs occurred in two stages, where the first stage, d1 started from 50 °C up to 330 °C and the second stages, d2 from 294 °C up to 447 °C, respectively. The first stages of the decomposition are mainly caused by the evaporation of solvent trapped inside the 3D-printed GPE and water present due to the hygroscopic nature of LiClO_4_. The second stage of decomposition is attributed to the decomposition of the PUA chain in the GPE. The increasing amount of LiClO_4_ in GPE shifted the onset degradation temperature for d2 to a lower temperature, as can be seen in Table 5. This occurrence may be due to the weakening of the C=O in the PUA chain caused by decreasing electron intensity as more Li^+^ ions interact with the oxygen atom [13]. The pure polymer decomposed entirely, leaving a residue of about 18.63%, whereas doped salt polymer left residues of around 22.13% to 31.41% of the weight. This residue increased in response to the addition of salt dopant while the PUA GPE stability improved as well. It means that more heat is needed to break down the polymer chain in the GPE as compared to pure PUA as the stronger intermolecular complexation between PUA polymer matrix with lithium salt [27].

### 2.7. Differential Scanning Calorimetry (DSC)

**Figure 12:**
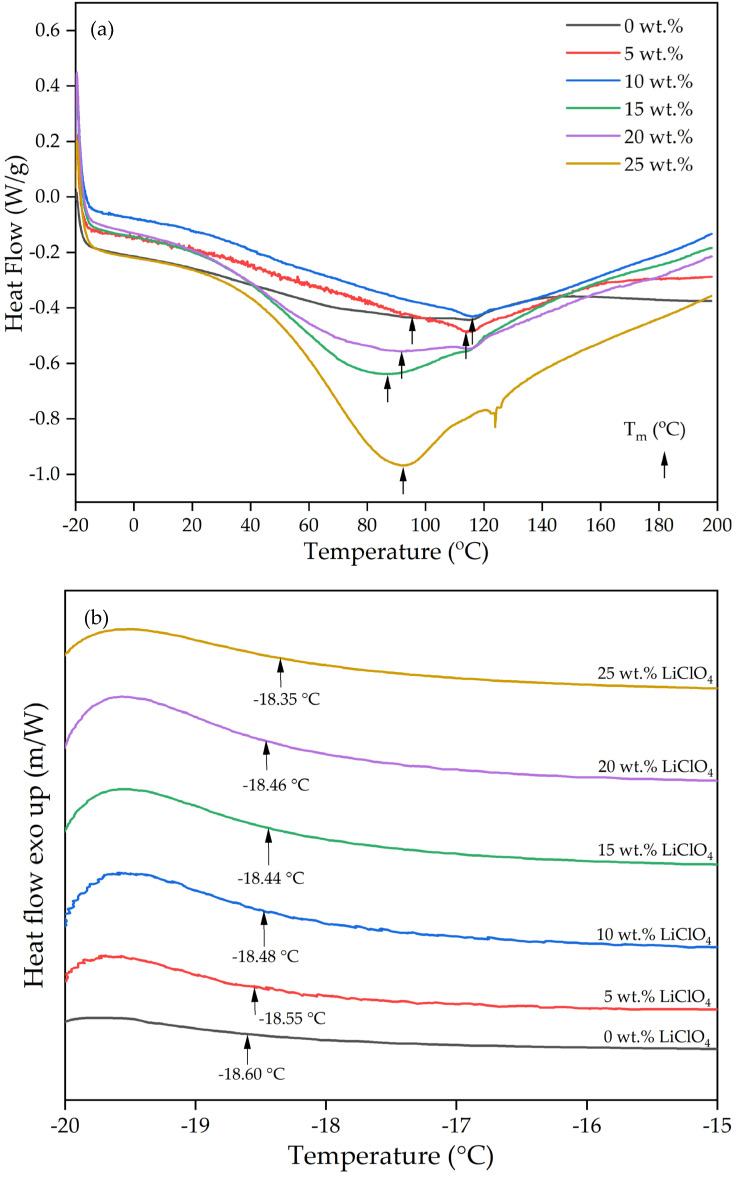
(**a**) DSC curves and (**b**) T_g_ values of 0 wt.% to 25 wt.% LiClO_4_ 3D-printed GPEs.

**Figure 13:**
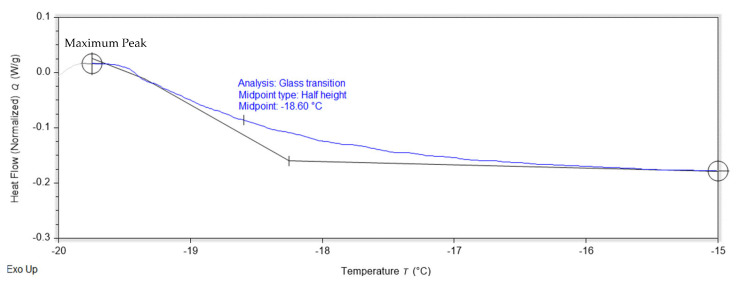
DSC analysis of 0 wt.% LiClO_4_ 3D-printed GPEs using TRIOS software.

The DSC thermograms of 3D-printed PUA-based GPE from 0 wt.% to 25wt.% of LiClO_4_ are shown in Figure 12 and Table 6. Based on Figure 12b, the transition glass temperature, T_g_ value for 0 wt.% was −18.60 °C, which is near to the PUA T_g_ value from a previous study [21]. All the samples were observed to have one T_g_ value and one endothermic melting peak, T_m_. Exothermic crystalline peaks were not detected, which indicates all the samples were amorphous, in line with the results in XRD. The T_g_ values were analyzed using TRIOS software from TA Instruments. The T_g_ was determined by the half-height midpoint temperature of heat flow changes. The range of the temperature change was taken from the heat flow maximum peak temperature to −15 °C as the obvious heat flow changes were observed within that range, as shown in Figure 13 below. Based on Table 6, the T_g_ values determined in this study were in a range with T_g_ from previous PUA GPE reports, which were from −20.4 °C up to −12.4 °C [21,43]. The addition of LiClO_4_ salt into the polymer increased the T_g_ value for all the samples. The increasing value of T_g_ compared to the undoped Li sample is due to two reasons. First, the coordination of Li ions with the polyether oxygen, where it partially arrests the segmental motion of the polymer segment, caused the higher number of cross links to occur. Second, the dipole–dipole interaction between Li ions with the polyether oxygen caused the PUA chain to stiffen. The result of increasing T_g_ with the addition of Li salt into the 3D-printed PUA-based GPE is in line with a previous study [15,44,45].

In addition, the addition of LiClO_4_ salt also increased the T_m_ in the samples up to 10 wt.% and decreased beyond it. This may be due to the interaction of free ions to the hydrogen bond in the -NH chain, which increased the strength of the intermolecular bond, thus, increasing the T_m_. Beyond 10 wt.%, the T_m_ decreased as less interaction between the -NH chain might be due to the growing number of ion pairs, which do not interact with the urethane chain. Basically, there are three main interactions that occur within the polymer electrolyte: (1) The interaction between ether oxygen with Li ions highlights the transient crosslinks between polyether chains, formed via Li ions, which limits the segmental motion. (2) The interaction between urethane -NH, carbonyl groups and Li ions, either intra- or inter-molecular crosslinking; (3) Interaction of mixed ether-urethane with Li ions, which leads to the mixing phase of hard and soft segments.

### 2.8. 3D-Printed PUA GPE with Different Geometries

**Figure 14:**
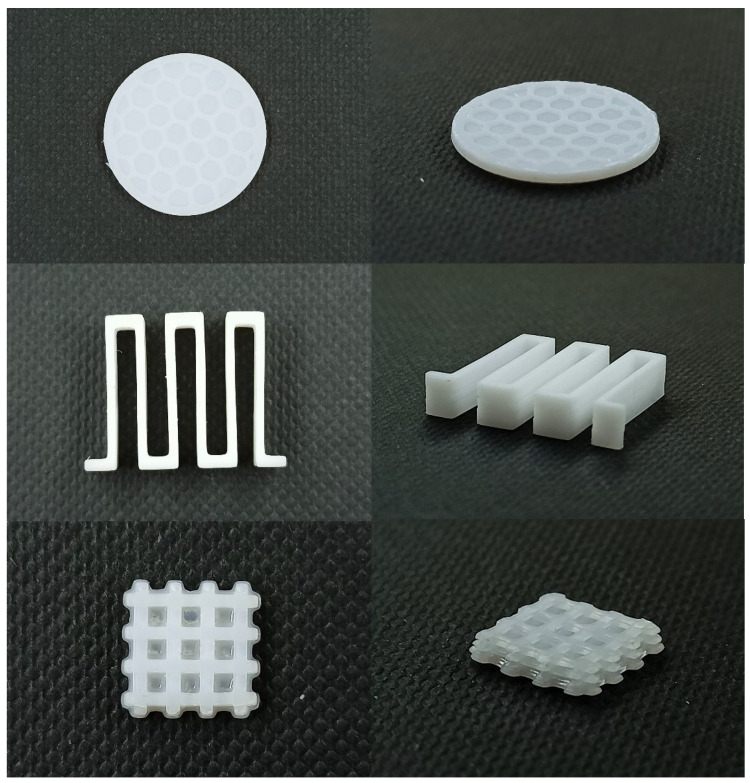
3D-printed PUA-based GPEs with different 3D geometries.

The 3D-printed GPEs were printed into three model designs, as shown in Figure 14. These shapes demonstrate the printability and capability of GPE fabrication into any design desired in order to improve battery performance using a 3D battery concept, especially at the research level.

## 3. Conclusions

3D-printed PUA-based GPEs with different concentrations of LiClO_4_ were prepared by using the 3D-printing stereolithography (SLA) method. The effects of each different LiClO_4_ concentration on conductivity and its correlation with changes in the functional group, morphology and transport number of 3D-printed PUA GPEs were investigated. The highest conductivity achieved was 1.24 × 10^−3^ S cm^−1^ at room temperature with samples consisting of 10 wt.% LiClO_4_. The conductivity of the 3DP GPEs were declined beyond 10 wt.%, which is correlated with the number of free ions from the analysis of FTIR deconvolution. Furthermore, the changes in the amorphous phase from the XRD measurement and the surface morphology of 3DP PUA GPEs indicated the presence of structural rearrangement of the polymer chain that leads to Li+ cation transportation. The FTIR studies show the band shifts of N-H, C=O and C-O-C functional groups, suggesting the LiClO_4_ complexation with an SLA PUA photopolymer. TNM analysis exhibiting high values demonstrated that the conductivity of the GPE is mainly contributed by ionic charges. Moreover, the optimum LiClO_4_ concentrations (10 wt.%) of PUA GPEs were successfully printed into 3D-structured GPEs. From this research, the PUA polymer displayed as an alternative material to be paired with stereolithography technology to produce high conductive polymer electrolytes with customization ability in the effort to maximize electrolyte performance.

## 4. Materials and Methods

### 4.1. Materials

Commercially available basic PUA resin was purchased from Anycubic Ltd., Shenzhen, Guangdong, China. Lithium perchlorate (LiClO_4_; ACS reagent, ≥95.0%) and dimethylformamide (DMF; ACS reagent, ≥99.8%) were obtained from Sigma Aldrich, St.Louis, MO, USA. All the materials were used as received without any purification process.

### 4.2. Methods

**Figure 15:**
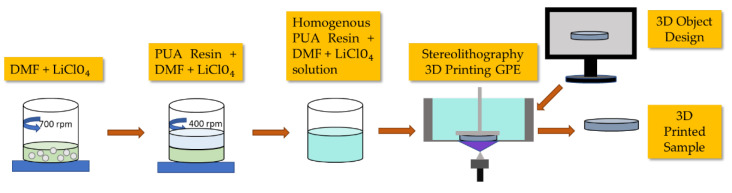
3D-printed PUA GPEs fabrication process.

**Figure 16:**
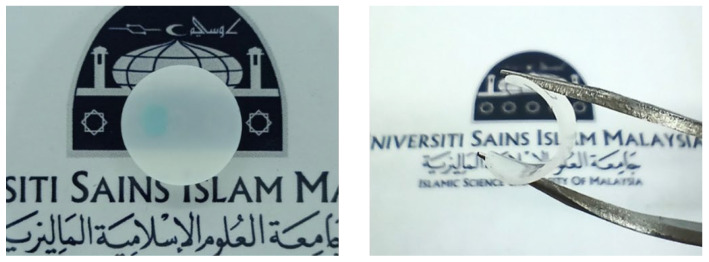
3D-printed GPE sample.

The 3D-printed PUA gel polymer electrolyte was prepared by using stereolithography method. First, various concentrations of LiClO_4_ (0–25 wt.%) as shown in Table 7 were dissolved in DMF and magnetically stirred at 60 °C for 30 min at 700 round per minute (rpm). Then, the salt solution was mixed with PUA resin and continued to be stirred at 400 rpm until a homogenous mixture was obtained. The concentration range was stopped at 25 wt.% LiClO_4_ because the printability factor of sample beyond it was poor due to high viscosity in the mixed resin which could lead to uneven surface uniformity and salt distribution within the sample. The homogenous mixture was then poured into a resin tray. A 3D SLA printer (Anycubic Photon S, Anycubic Ltd., Shenzhen, Guangdong, China) with 405 nm UV integrated light was used to print the designed film samples using printer setting as shown in Table 8. The UV emitted from the 3D printer induced polymerization process that transformed the electrolyte mixture into GPE samples. Anhydrous isopropyl alcohol was used to remove excess uncured electrolyte solution from the GPE. The prepared 3D-printed GPEs were placed in a desiccator overnight before undergoing further characterizations. Figure 15 shows the preparation process of 3D-printed PUA GPEs and the 3D-printed PUA GPEs are shown in Figure 16.

### 4.3. Characterizations

The electrical properties of 3D-printed PUA GPEs were determined from electrochemical impedance spectroscopy (EIS). Hioki 3532-50 LCR Hi-tester (Nagona, Japan) was used to measure the impedance of 3D-printed GPEs at room temperature. The printed GPE was sandwiched between two round-shape stainless-steel electrodes wired to the instrument. An alternating voltage of 10 mV with frequency from 5 MHz to 50 Hz was applied across the sample. The current through the sample was measured and the impedance was deduced. From the impedance data obtained, the ionic conductivity (σ) was calculated using Equation (2).

(2) $$\sigma =\frac{t}{R_{b}A}$$

In Equation (2), *t* refers to the thickness of 3D-printed GPE (0.05 cm), *R_b_* refers to the bulk resistance and *A* refers to the area of the film in contact with the electrode (2.54 cm^2^).

From the impedance data, the charge carrier number density ( n) , mobility ( μ) and diffusion coefficient (D) were determined using Equations (3)–(5) accordingly [34].

(3) $$n=\left(\frac{m_{LiClO_{4}}}{M_{W}}\times N_{A}\right)\frac{1}{V_{T}}\times \%f_{ions}\times 2$$

(4) $$\mu =\frac{\sigma }{ne}$$

(5) $$D=\frac{\mu K_{b}T}{e}$$

Here $m_{LiClO_{4}}$ = mass of LiClO_4_, $M_{W}$ = molecular weight, $V_{T}$ = total volume of GPE, e = electron charges, $K_{b}$ = Boltzmann constant, *T* = temperature (in Kelvin) and %f_ions_ = percentage of free ions determined from FTIR deconvolution. In addition, the dielectric constant ( $\varepsilon_{r})$ , dielectric loss ( $\varepsilon_{i})$ , real electrical modulus ( $M_{r}$ ), imaginary electrical modulus ( $M_{i})\;$ and tan δ were calculated using these following equations [25,36]:

(6) $$\varepsilon_{r}=\frac{Z_{r}}{\omega C_{o}\left(Z_{r}^{2}+Z_{i}^{2}\right)}$$

(7) $$\varepsilon_{i}=\frac{Z_{i}}{\omega C_{o}\left(Z_{r}^{2}+Z_{i}^{2}\right)}$$

(8) $$M_{r}=\frac{\varepsilon_{r}}{\varepsilon_{r}^{2}+\varepsilon_{i}^{2}}$$

(9) $$M_{i}=\frac{\varepsilon_{i}}{\varepsilon_{r}^{2}+\varepsilon_{i}^{2}}$$

(10) $$\tan\delta =\frac{Z_{r}}{Z_{i}}$$

FTIR spectroscopy were carried out using Thermo Fisher Scientific model Nicolet iS 50 (Waltham, MA, USA) in order to confirm the complexation between polymer, salt and plasticizer. The measurement was performed at room temperature at a wavenumber between 4000 and 500 cm^−1^ with scanning resolution of 4 cm^−1^. To evaluate the percentage of free ions and ion pairs in the GPE system, the FTIR spectrum between 600 and 650 cm^−1^ was deconvoluted using Origin software. From that, the area under the graph of the free ions and ion pairs were calculated using the equations below [34]:

(11) $$f_{ions}\left(\%\right)=\frac{A_{f}}{A_{f}+A_{P}}\times 100$$

(12) $$P_{ions}\left(\%\right)=\frac{A_{p}}{A_{f}+A_{P}}\times 100$$

In Equations (11) and (12), *A_f_* is the area of free ions and *A_p_* is the area of ion pairs.

The XRD patterns of the samples were obtained by using Rigaku Mini Flex 600 diffractometer (The Woodlands, TX, USA) to understand the structure properties of 3D-printed GPE. The measurement was performed at ambient temperature with scanning angle of 2θ from 10° to 60° at 0.004o/S and X-ray radiation wavelength of 1.5406Å.

The sample’s transference number was determined using the DC polarization technique. The DC current was measured as a function of time using a constant DC potential of 1.5V across the SS/3D-printed GPE/SS cell and recorded with the UT803 interface program. The stainless steel (SS) acts as an ion-blocking electrode. The electronic and ionic transference numbers were calculated using the equations below [41]:

(13) $$t_{e}=\frac{\sigma_{e}}{\sigma_{t}}=\frac{i_{e}}{i_{t}}$$

(14) $$t_{i}=1-\frac{i_{e}}{i_{t}}=1-t_{e}\;$$

Here $t_{e}$ and $t_{i}$ are the electronic and ionic transference numbers, respectively. The $\sigma_{e}$ and $\sigma_{t}$ refer to the electronic and total conductivities, respectively.

DSC 250 (New Castle, DE, USA) was used to study the phase transition of the polymer electrolyte during the heating process. The study was conducted between −20 °C and 150 °C, at 10 °C/min scanning rate under nitrogen atmosphere at 10 mL/min flow rate. Thermogravimetric analysis (TGA) was operated using TGA 550 (New Castle, DE, USA) from 50 °C to 600 °C with a heating rate of 10 °C/min in a dynamic nitrogen atmosphere at 20 mL/min flow rate.

Surface morphology of 3D-printed PUA electrolyte was investigated by scanning electron microscopy (SEM) model JEOL JSM-7600F (Singapore).

## Figures and Tables

**Table 1:** Percentage of free ions and ion pairs of 3D-printed PUA-based GPE with different LiClO_4_ concentrations.

LiClO_4_ Content (wt.%)	Free Ion (%)	Ions Pair (%)
5	94.98	5.02
10	99.90	0.10
15	98.23	1.77
20	92.09	7.91
25	90.94	9.06

**Table 2:** Bulk resistance and ionic conductivity of SLA-printed PUA-based GPE system.

LiClO_4_ (wt.%)	R_b_ (Ω)	σ (S cm^−1^)
0	59874	3.11 × 10^−7^
5	68.38	2.72 × 10^−4^
10	14.95	1.24 × 10^−3^
15	21.06	8.83 × 10^−4^
20	30.71	6.06 × 10^−4^
25	38.09	4.88 × 10^−4^

**Table 3:** The conductivity of PUA GPEs from previous study.

PUA Electrolyte System	Conductivity (S cm^−1^)	References
67 wt.% PUA, 33 wt.% 1 M LiClO_4_ in PC.	4.00 × 10^−4^	[33]
77.71 wt.% PUA, 15 wt.% LiClO_4_, 4.49 wt.% TMPTA, 2.80 w.t% darocure.	8.96 × 10^−5^	[34]
16.2 wt. % PUA, 19 wt. %DEEYTFSI,6.61 wt. % LiTFSI, 52.90 w.t % MMA, 5 wt. %EDGMA.	2.76 × 10^−4^	[35]
38.5 wt. % PUA, 38.5 wt. % PC, 23 wt.% LiClO_4_.	3.7 × 10^−3^	[15]

**Table 4:** Maximum peak of tan δ and relaxation time (τ) of the GPE derived from Figure 5e.

LiClO_4_ (wt.%)	Maximum Peak (Hz)	τ (s)
5	4.20	9.95 × 10^−6^
10	6.40	6.37 × 10^−8^
15	6.04	1.45 × 10^−7^
20	4.85	2.27 × 10^−6^
25	4.97	1.71 × 10^−6^

**Table 5:** Analysis of TG of 3D-printed GPE PUA.

LiClO_4_ Content	Decomposition Stages	Degradation Temp (°C)		Weight Loss (%)	Residue (%)
		Onset X	End X		
0 wt.%	d1	-	-	82.37	18.63
	d2	396.98	447.68		
5 wt.%	d1	59.80	330.17	77.87	22.13
	d2	330.17	419.44		
10 wt.%	d1	60.30	301.30	73.71	23.04
	d2	302.39	382.32		
15 wt.%	d1	58.20	294.00	72.72	27.28
	d2	294.76	336.95		
20 wt.%	d1	57.50	289.05	69.21	30.79
	d2	299.45	352.05		
25 wt.%	d1	51.49	302.12	68.59	31.41
	d1	304.5	332.98		

**Table 6:** Transition glass and melting temperature of 3D-printed GPE PUA.

LiClO_4_ Content	T_g_ (°C)	T_m_ (°C)
0 wt.%	−18.60	95.01
5 wt.%	−18.55	114.00
10 wt.%	−18.48	115.85
15wt.%	−18.44	86.30
20 wt.%	−18.46	91.55
25 wt.%	−18.35	92.77

**Table 7:** 3D-printed PUA GPEs composition ratio.

LiClO_4_ Concentration (wt.%)	Resin (g)	LiClO_4_ (g)	Solvent (g)
0	1	0.00	0.75
5	1	0.09	0.75
10	1	0.19	0.75
15	1	0.31	0.75
20	1	0.44	0.75
25	1	0.58	0.75

**Table 8:** 3D printer settings parameter.

Layer (cm)	Exposure Time (s)	Off Time (s)
0.05	10	4
