# Correction: Norjeli et al. Additive Manufacturing Polyurethane Acrylate via Stereolithography for 3D Structure Polymer Electrolyte Application. *Gels* 2022, *8*, 589

**DOI:** 10.3390/gels10070423

**Published:** 2024-06-27

**Authors:** Muhammad Faishal Norjeli, Nizam Tamchek, Zurina Osman, Ikhwan Syafiq Mohd Noor, Mohd Zieauddin Kufian, Mohd Ifwat Bin Mohd Ghazali

**Affiliations:** 1 SMART RG, Faculty of Science and Technology, Universiti Sains Islam Malaysia, Nilai 71800, Malaysia; faishal5453@raudah.usim.edu.my; 2 Department of Physics, Faculty of Science, Universiti Putra Malaysia, Serdang 43400, Malaysia; nizamtam@upm.edu.my; 3 Centre for Ionics Universiti Malaya, Department of Physics, Faculty of Science, Universiti Malaya, Kuala Lumpur 50603, Malaysia; zurinaosman@um.edu.my (Z.O.); mzkufian@um.edu.my (M.Z.K.); 4 Physics Division, Centre of Foundation Studies for Agricultural Science, Universiti Putra Malaysia, Serdang 43400, Malaysia; imnoor@upm.edu.my

In the original publication [1], there was a mistake in Figure 11 as published. There was a mistake on the y-axis of the graph in Figure 11. The corrected Figure 11 appears below.

The authors state that the scientific conclusions are unaffected. This correction was approved by the Academic Editor. The original publication has also been updated.

## Figures and Tables

**Figure 11:**
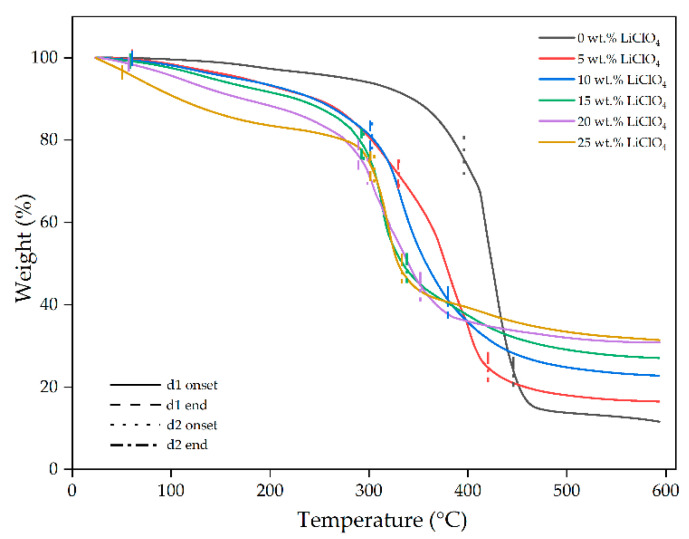
Thermograms of 3DP GPE PUA with different wt.% of LiClO_4_.
